# Development and validation of a population-based prognostic nomogram for primary colorectal lymphoma patients

**DOI:** 10.3389/fonc.2022.991560

**Published:** 2022-10-24

**Authors:** Qian Chen, Yang Feng, Jiaxin Yang, Rui Liu

**Affiliations:** ^1^ Department of Oncology, The Second Affiliated Hospital of Chongqing Medical University, Chongqing, China; ^2^ Department of Breast and Thyroid Surgery, The Second Affiliated Hospital of Chongqing Medical University, Chongqing, China

**Keywords:** primary colorectal lymphoma, overall survival, nomogram, SEER database, prognostic

## Abstract

**Background:**

Primary colorectal lymphoma (PCL) is a relatively rare cancer type, constituting 15%–20% of primary gastrointestinal lymphoma and <1% of all colorectal malignancies. Given its low incidence, standard guidelines for case management are not available. This large population-based study aims to construct a nomogram to predict survival outcomes and to help tailor individualised treatment decisions in patients with PCL.

**Methods:**

A retrospective cohort study of patients with PCL was developed using data registered in the Surveillance, Epidemiology, and End Results (SEER) database between 1990 and 2015. The prognostic nomogram was constructed using R software after univariate and multivariate Cox regression analyses. Cox regression models were assessed using the proportional hazards (PH) assumption. Kaplan−Meier survival analysis was used to analyze survival outcomes. The 1-, 3-, 5-, and 10-year area under the curve (AUC) values of ROC (receiver operating characteristic) curves, the concordance index (C-index), and calibration curves were calculated to verify the predictive performance of the nomogram.

**Results:**

The final nomogram included age, Ann Arbor stage, histology, location, marital status, and treatment, all of which had an important effect on overall survival (OS). The discrimination of the nomogram revealed good prognostic accuracy and clinical applicability as indicated by C-index values of 0.713 and 0.711 in the training and validation cohorts, respectively. Kaplan−Meier survival curves were significantly different for distinct conditions.

**Conclusion:**

This study developed and validated a six-factor nomogram for predicting PCL patient prognosis. This nomogram might be useful for risk stratification and making better individualised decisions for PCL patients.

## Introduction

Approximately 40% of lymphomas have extranodal manifestations, and the gastrointestinal tract (GIT) is the most common site for extranodal lymphoma (1). In the GIT, the most common organs of involvement include the stomach (50%–60%) and small intestines (20%–30%) (2). Primary colorectal lymphomas are rare, constituting only 10%–20% of gastrointestinal lymphomas and 0.2%–0.6% of large intestine neoplasms (3). For colorectal lymphomas, it was reported that the caecum was the most common location (4), perhaps due to the presence of abundant lymphoid tissue (5). Histopathologically, the most common histological subtype of lymphoma involving the GIT is diffuse large B-cell lymphoma (DLBCL), followed by mucosal-associated lymphoid tissue (MALT) lymphoma, and T-cell lymphomas are very rare (6). Many patients with primary colorectal lymphoma have non-classical clinical symptoms in the early stage. The first presentation includes abdominal pain (40%–90%), and the second is weight loss (3). Other rare presentations include anorexia or obstruction, abdominal mass, diarrhea, nausea and vomiting, perforation, peritonitis signs, and bleeding loss (7). Owing to non-specific symptoms, the diagnosis is often delayed.

The original Dawson’s criteria for primary gastrointestinal lymphoma included (1) the absence of clinically enlarged lymph nodes on physical examination, (2) the absence of enlarged mediastinal lymph nodes on chest radiography, (3) normal hematological laboratory values and bone marrow biopsy, and (4) no lymphomatous involvement of the liver and spleen, all in the setting of lymphoma occurring in a portion of the gastrointestinal tract (5). With the development of computed tomography, these criteria have been expanded with the requirement that retroperitoneal or mediastinal lymphadenopathy be excluded.

The treatment strategies for gastrointestinal lymphoma include surgery, chemotherapy, radiotherapy, and immunotherapy, or their combinations. Combination chemotherapy with rituximab, cyclophosphamide, doxorubicin, vincristine, and prednisolone (R-CHOP) has become the baseline and gold standard for treating PCLs. However, the role of surgery in treating colonic lymphomas remains controversial. Surgery is typically used for emergency circumstances, such as obstruction, perforation, or bleeding (8). Some professionals suggested surgery as the preliminary means of treatment because it could defend against complications and increase the probability of cure with or without adjuvant chemotherapy (9, 10). Moreover, radiation therapy has certain limitations in terms of complications associated with therapy and limited control of PCLs. In recent years, the incidence of colorectal lymphoma has increased. Notably, the learning regarding the clinical prediction of PCL is limited given the very few studies focusing on nomogram effectuation. In this study, a PCL-targeting nomogram was established for prognostic prediction based on a large sample size retrieved from the Surveillance, Epidemiology, and End Results (SEER) database. The nomogram was developed to visualize the prognostic strength of the different factors from the multivariate Cox regression model in a single diagram and used to facilitate better risk stratification and tailor treatment decisions. After the retrospective analysis of PCL patients, a new prognostic nomogram was conducted to screen out the risk factors and different treatment on affecting the OS of PCL patients, which could help doctors identify “medium-risk” and “high-risk” patients and optimize therapy strategies.

## Materials and methods

### Data source

We retrieved clinicopathological data and prognostic results of PCL cases from 199 to 2015 in the Surveillance, Epidemiology, and End Results (SEER) database of the National Cancer Institute (http://seer.cancer.gov/), which represents approximately 30% of the total American population (11). The included data conformed to the following criteria: (I) the International Classification of Diseases (ICD) code O-3 was used to identify PCLs by site codes (C18.0, C18.2, C18.3, C18.4, C18.5, C18.6, C18.7, C18.9, C19.9, and C20.9) and morphology code; (II) PCL as the only or first primary cancer that was confirmed by histology; (III) lymphoma histology was diffuse large B-cell lymphoma (DCBCL), follicular lymphoma (FL), extranodal marginal zone lymphoma (MZL) of mucosal-associated lymphoid tissue (MALT), or mantle cell lymphoma (MCL). The exclusion criteria were as follows: (I) absence of histological confirmation result, unknown tumor-directed surgery or radiation, and unknown clinicopathological information, including marital status, American Joint Committee on Cancer (AJCC) stage, race type, and survival month.

### Study variables

We examined clinical variables from the SEER*8.3.9 program, including age at diagnosis, race, sex, marital status, primary site, histological type, AJCC stage, surgery, chemotherapy, radiotherapy, survival months, and survival status. The definition of each enrolled variable was as follows: 1) race was classified as white, black, and other (defined as American Indian/AK Native, Asian/Pacific Islander); 2) sex was divided into female and male; 3) the diagnosis of year was divided into three groups, namely, 1990–1999, 2000–2009, and 2010–2015; 4) the locations of primary lymphoma were divided into the right (located to the caecum, ascending colon, hepatic flexure of colon, transverse colon, and splenic flexure of colon), the left (located to the descending colon and sigmoid colon), the rectum (including rectum and rectosigmoid junction), and NOS (not otherwise specified in the colon); 5) the histological types of cancers were divided into four groups, including DLBCL, MZL, MCL, and FL; 6) the age of PCLs was set as a continuous variable; 7) marital status at diagnosis was divided into two groups, namely, married and others (including divorced, separated, single, widowed, unmarried, and domestic partner); 8) the Ann Arbor stage was divided into stages I, II, III, and IV; and 9) treatment was classified as i) no surgery or chemotherapy or radiation (no), ii) chemotherapy only (c), iii) radiation only (r), iv) radiation combined chemotherapy (r+c), v) surgery only (s), vi) surgery combined chemotherapy (s+c), vii) surgery combined radiation (s+r), or viii) surgery combined radiation and chemotherapy (s+r+c). The outcome of interest was overall survival (OS). OS was defined as the time from the diagnosis of PCL to death attributed to any cause.

### Statistical analysis

All statistical analyses were performed using R software (R Foundation, Vienna, Austria, version 3.5.3, http://www.r-project.org). A p-value of <0.05 was defined as the statistical significance for variable extraction when performing backwards stepwise selection. These independent factors were identified by the results of univariate and multivariate Cox regression analyses. The hazard ratios (HRs) were calculated. Each factor exhibited a correlation with OS, and a nomogram was developed according to the coefficients by the “rms” package (12). Based on the results of multivariate cox regression analysis, clinicopathological factor-based nomogram was developed by using RMS package. Each variable corresponded to a specific point, and these points add up to the total point. We could estimate the 1, 3, 5, and 10-year overall survival rate for PCL patients by drafting a vertical line between the total point axis and each of the three prognosis axes. Receiver operating characteristic (ROC) curves were generated to estimate the clinical applicability of the model, and calibration plots were used to verify whether the predicted survival and actual survival were in concordance. Survival analysis was performed to define the prognostic factors based on the Cox model. X-tile software was applied to divide patients into low-, medium-, and high-risk groups. Kaplan−Meier survival analysis was used to compare the prognosis among different risk stratification groups and different clinicopathological PCL cases.

## Results

### Baseline characteristics

A total of 2,350 patients were identified with PCL based on histology and randomly divided into a training cohort (1,646 patients) and a validation cohort (704 patients) at a ratio of 7:3. The demographic and clinical characteristics of these PCL cases are generalized in **Table 1**. Among all the cases, the median age at presentation was 64.6 years (range, 2.0–99.0 years). The following characteristics were noted in the majority of the PCL population: Caucasian ethnicity (81.7%), men (61.3%), married (61.4%), localized on the right side (56.2%), histologically confirmed as DCBCL (57.8%), and stage I disease (46.3%). With regard to treatment, approximately 14.1% of affected patients did not receive surgery, chemotherapy, or radiation, and 22.7% of cases exclusively underwent chemotherapy. A total of 2.4% of cases only underwent radiation. Additionally, 3.0% of patients received chemotherapy combined with radiation, 26.4% of patients received surgery combined with chemotherapy, and 1.2% of patients received surgery combined with radiation. Furthermore, 1.7% of patients received surgery, radiation, and chemotherapy. According to our statistics, from 1990 to 1999, the main treatment methods for colorectal lymphoma patients were surgery combined chemotherapy (36.9%), surgery only (28.3%), and chemotherapy only (19.5%). Between 2000 and 2009, the main treatment strategies were surgery only (29.0%), surgery combined chemotherapy (25.6%), and chemotherapy only (22.4%). Between 2010 and 2015, the main treatment options were surgery only (27.3%), chemotherapy only (25.1%), and surgery combined chemotherapy (21.4%).

**Table 1:** Baseline demographic and clinical characteristics of PCL patients.

	Overall	Training cohort	Validation cohort	p-value
	(n=2,350)	(n=1,646)	(n=704)	
**Race (%)**				
White	1,920 (81.7)	1,336 (81.2)	584 (83.0)	0.12
Black	143 (6.1)	95 (5.8)	48 (6.8)	
Other	287 (12.2)	215 (13.1)	72 (10.2)	
**Gender (%)**				
Female	909 (38.7)	633 (38.5)	276 (39.2)	0.77
Male	1,441 (61.3)	1,013 (61.5)	428 (60.8)	
**Year (%)**				
1990–1999	409 (17.4)	281 (17.1)	128 (18.2)	0.81
2000–2009	1,261 (53.7)	886 (53.8)	375 (53.3)	
2010–2015	680 (28.9)	479 (29.1)	201 (28.6)	
**Location (%)**				
Right	1,321 (56.2)	938 (57.0)	383 (54.4)	0.65
Left	350 (14.9)	237 (14.4)	113 (16.1)	
Nos	287 (12.2)	199 (12.1)	88 (12.5)	
Rectum	392 (16.7)	272 (16.5)	120 (17.0)	
**Histology (%)**				
DCBCL	1,359 (57.8)	939 (57.0)	420 (59.7)	0.33
MZL	489 (20.8)	353 (21.4)	136 (19.3)	
MCL	250 (10.6)	183 (11.1)	67 (9.5)	
FL	252 (10.7)	171 (10.4)	81 (11.5)	
**Age (mean (SD))**	64.6 (15.9)	64.5 (15.9)	64.7 (16.0)	0.75
**Marital_status (%)**				
Married	1,444 (61.4)	1,009 (61.3)	435 (61.8)	0.86
Other	906 (38.6)	637 (38.7)	269 (38.2)	
**AnnArbor_Stage (%)**				
I	1,087 (46.3)	754 (45.8)	333 (47.3)	0.22
II	555 (23.6)	376 (22.8)	179 (25.4)	
III	139 (5.9)	103 (6.3)	36 (5.1)	
IV	569 (24.2)	413 (25.1)	156 (22.2)	
**Treatment (%)**				
No	331 (14.1)	233 (14.2)	98 (13.9)	0.94
Chemotherapy Only	534 (22.7)	374 (22.7)	160 (22.7)	
Radiation Only	57 (2.4)	40 (2.4)	17 (2.4)	
R+C	71 (3.0)	49 (3.0)	22 (3.1)	
Surgery Only	668 (28.4)	467 (28.4)	201 (28.6)	
S+C	621 (26.4)	440 (26.7)	181 (25.7)	
S+R	29 (1.2)	17 (1.0)	12 (1.7)	
S+R+C	39 (1.7)	26 (1.6)	13 (1.8)	

DCBCL, diffuse large B-cell lymphoma; FL, follicular lymphoma; MCL, mantle cell lymphoma, MZL, extranodal marginal zone lymphoma of mucosal-associated lymphoid tissue; Nos, not otherwise specified in colon.

S+C, surgery combined with chemotherapy; S+R, surgery combined with radiation.

C+R, chemotherapy combined with radiation; S+C+R, surgery combined with chemotherapy and radiation.

### Survival analysis

**Figure 1:**
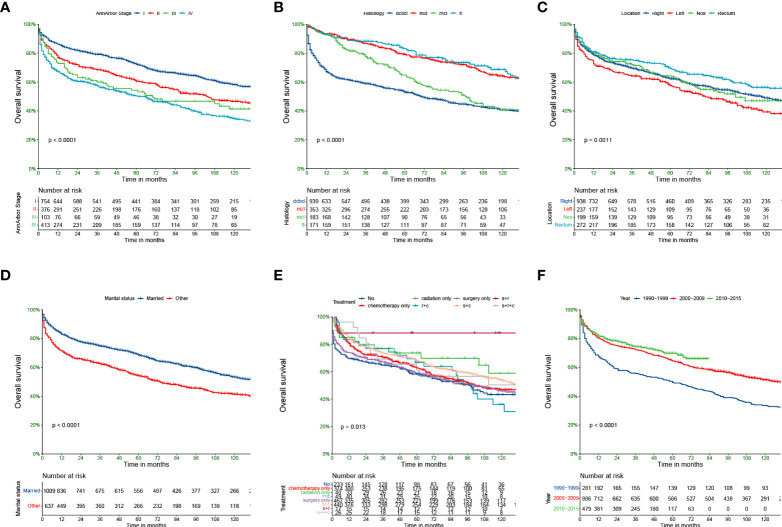
Kaplan-Meier survival analysis for PCL patients. AnnArbor stage **(A)**, histology **(B)**, Location **(C)**, marital status **(D)**, Treatment **(E)**, Diagnosis of year **(F)**.

The Kaplan—Meier curves for the main subtypes are shown in **Figure 1**. The results demonstrated that the earlier the tumour stage, the better the prognosis. The median survival rates of stages I–IV PCL patients were 160, 103, 70, and 61 months. The median survival rate of patients with MZL was the longest (164 months), and the median survival rate of those with DCBCL (70 months) was the shortest. In terms of the site of lymphoma, the median survival rates of those with the right-side disease, left-side disease, NOS, and rectal disease were 117, 82, 101, and 149 months, respectively. The prognoses of younger patients and married patients were better than those of other patient subgroups. In terms of treatment strategies, patients who underwent the combination of surgery, radiotherapy and chemotherapy had better OS.

### Univariate and multivariate cox regression analysis

**Figure 2:**
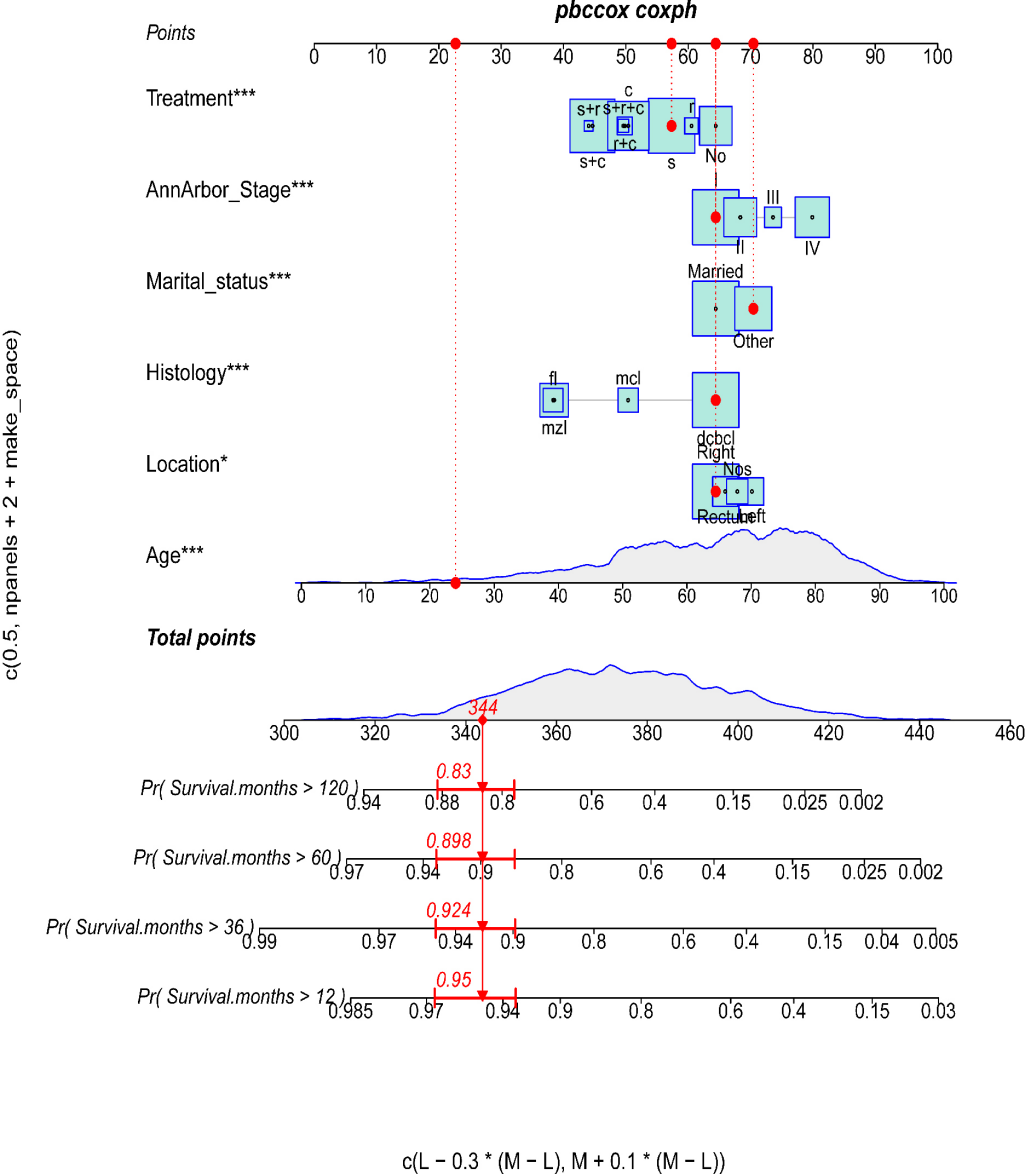
The predictive nomogram for OS of PCL patients. The symbols “*” indicate that the p-value for this variable is less than 0.05. More symbols “*” indicate a smaller p-value.

The proportional-hazards assumption was evaluated and found reasonable for each variable. These results of PH assumption for Cox regression were as follows: the PH test (continuous_age) may slightly violate, but the results fit data in general. Analyses were stratified by histology, the result of histology_MZL,MCL.FL was appreciable (**Supplementary Material**). Univariate Cox regression analyses revealed that age (p<0.01), Ann Arbor stage (p<0.01), histology (p<0.01), location (p=0.01), marital status (p<0.01), year of diagnosis (p<0.01), and treatment (p<0.01) were remarkably associated with overall survival (**Table 2**). Multivariable Cox regression analyses revealed that age (p<0.01); Ann Arbor stage III (p<0.01) and IV (p<0.01); FL (p<0.01), MCL (p<0.01), and MZL (p<0.01) histology; left-side disease (p=0.02); other marital status (p<0.01); and c (p<0.01), r+c (p<0.01), s+c (p<0.01), and s (p<0.01) treatment were included in the final nomogram model (**Figure 2**). We found that a diagnosis in 2000–2009 (p<0.01) and 2010–2015 (p<0.01) affected OS.

**Table 2:** Univariate and multivariate Cox regression analysis of OS.

	Univariate Cox regression		Multivariate cox regression	
Characteristics	HR.CI95	p-value	HR.CI95	p-value
**Age**	1.05 (1.04–1.05)	<0.01	1.04 (1.04–1.05)	<0.01
**Ann Arbor stage**				
I	Reference		Reference	
II	1.42 (1.19–1.7)	<0.01	1.15 (0.95–1.39)	0.16
III	1.65 (1.24–2.19)	<0.01	1.52 (1.14–2.05)	<0.01
IV	2.06 (1.74–2.43)	<0.01	1.87 (1.56–2.24)	<0.01
**Gender**				
Female	Reference		Reference	
Male	1.1 (0.96–1.27)	0.18	NA	NA
**Histology**				
DCBCL	Reference		Reference	
FL	0.39 (0.29–0.52)	<0.01	0.35 (0.26–0.47)	<0.01
MCL	0.76 (0.61–0.95)	0.02	0.57 (0.45–0.73)	<0.01
MZL	0.45 (0.37–0.54)	<0.01	0.35 (0.28–0.44)	<0.01
**Location**				
Right	Reference		Reference	
Left	1.29 (1.06–1.56)	0.01	1.26 (1.03–1.53)	0.02
Nos	0.97 (0.77–1.22)	0.81	1.21 (0.95–1.54)	0.12
Rectum	0.78 (0.63–0.95)	0.02	1.05 (0.84–1.31)	0.68
**Marital status**				
Married	Reference		Reference	
Other	1.42 (1.23–1.63)	<0.01	1.29 (1.12–1.49)	<0.01
**Race**				
White	Reference		Reference	
Black	0.84 (0.61–1.15)	0.27	1.1 (0.79–1.51)	0.58
Other	0.74 (0.6–0.92)	<0.01	0.85 (0.68–1.06)	0.14
**Treatment**				
No	Reference		Reference	
Chemotherapy only	0.82 (0.65–1.04)	0.10	0.55 (0.43–0.71)	<0.01
R+C	0.94 (0.62–1.43)	0.78	0.51 (0.33–0.79)	<0.01
Radiation only	0.67 (0.4–1.13)	0.14	0.83 (0.49–1.42)	0.50
S+C	0.71 (0.57–0.89)	<0.01	0.41 (0.32–0.53)	<0.01
S+R	0.3 (0.11–0.81)	0.02	0.4 (0.15–1.1)	0.08
S+R+C	0.65 (0.36–1.17)	0.15	0.55 (0.3–1.02)	0.06
Surgery only	0.92 (0.74–1.15)	0.47	0.69 (0.55–0.88)	<0.01
**Year**				
1990–1999	Reference		Reference	
2000–2009	0.63 (0.53–0.74)	<0.01	0.68 (0.57–0.81)	<0.01
Year2010–2015	0.53 (0.42–0.66)	<0.01	0.54 (0.43–0.68)	<0.01

HR, hazard ratio; CI, confidence intervals; NA, not applicable.

### Construction and validation of the prognostic nomogram

**Figure 3:**
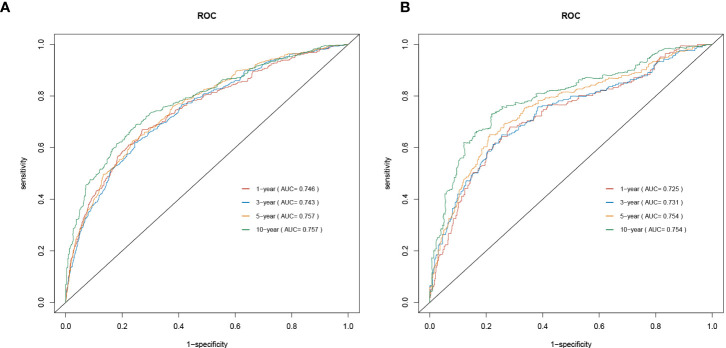
The 1,3,5,10-year ROC curves for training **(A)** and validation **(B)** cohort.

**Figure 4:**
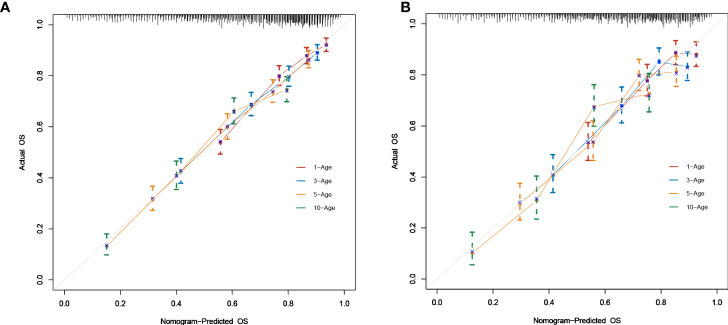
The 1,3,5,10-year Calibration curves for training **(A)** and validation **(B)** cohort.

As shown in **Figure 2**, a prognostic nomogram that included six independent prognostic factors indicated by the multivariable Cox regression analysis was established to predict 1-, 3-, 5-, and 10-year OS for PCL patients. In the nomogram, individual patients were assigned a score for each variable axis according to the patient’s physical circumstances. The sum of these points was located on the total points scale. Then, we estimated the probability of 1-, 3-, 5-, and 10-year overall survival. We further performed internal validation by randomly dividing the patients into training and validation groups at a ratio of 7:3. We evaluated the predictive ability of the nomogram using ROC curves. Area under the curve (AUC) values represent the predicted probability of the model (AUC=0.5, absent; AUC=0.5–0.7, negative; AUC=0.7–0.9, satisfactory; AUC>0.9, good) (13). In the training cohort, the 1-, 3-, 5-, and 10-year area under the curve (AUC) values of the nomogram for OS were 0.746, 0.743, 0.757, and 0.757, respectively (**Figure 3A**). In the validation cohort, the 1-, 3-, 5-, and 10-year AUCs were 0.725, 0.731, 0.754, and 0.754, respectively (**Figure 3B**). Harrell’s concordance index (C-index) was also generated to assess the prognostic values of the nomogram. The C-index for the nomogram for the prediction of OS was 0.713 in the training dataset and 0.711 in the validation dataset, indicating the stability and effectiveness of the established nomogram. Moreover, calibration curves (**Figures 4A, B**) also showed that the nomogram had considerable discriminative abilities for predicting OS in both datasets.

### Nomogram performance in stratifying risk

**Figure 5:**
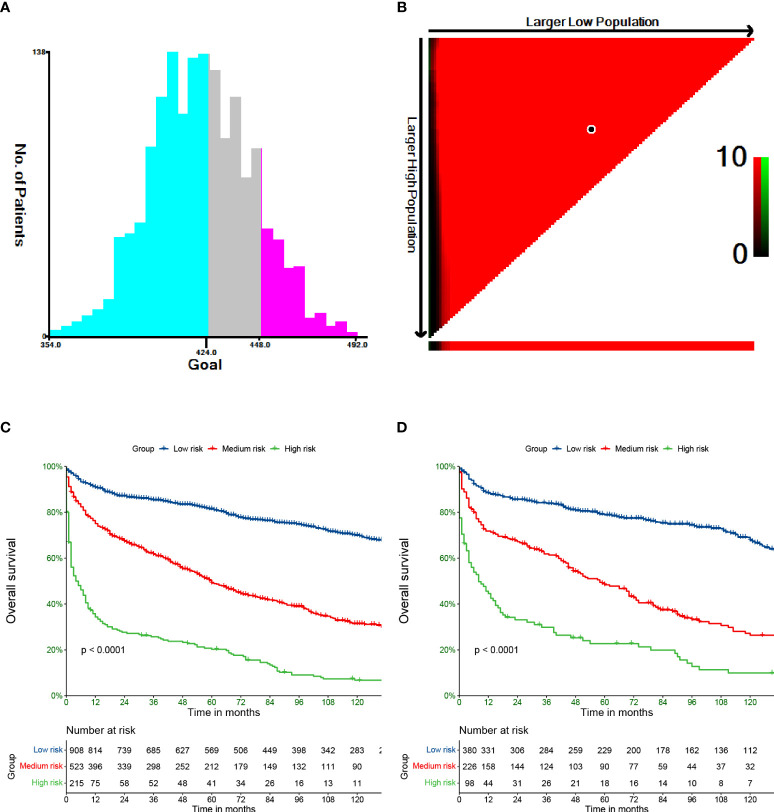
Cut-off values calculated by X-tile **(A, B)**. Overall survival of PCL stratified by risk in training **(C)** and validation **(D)** cohort.

We used X-tile software to divide patients into low-, medium-, and high-risk groups. The cutoff points were 424 and 448 (**Figures 5A, B**). Additionally, in the training set, 908, 523, and 215 patients were classified into the low-, medium-, and high-risk groups, respectively (p<0.0001). The low-, medium-, and high-risk groups included 380, 226, and 98 patients in the validation set. The high-risk patients had the worst OS, and the low-risk patients had the best OS based on Kaplan−Meier analyses (**Figures 5C, D**).

## Discussion

PCL is an extremely rare malignant tumor, accounting for 0.2%–0.6% of colorectal neoplasms. The condition is difficult to diagnose given its low incidence and the variability in histopathology. Given the lack of big data analyses of this disease, the correlative overall survival factors remain controversial, and treatment guidelines are not available. Therefore, we developed a nomogram that included six variables: age, histology, Ann Arbor stage, marital status, location, and treatment. The validation of the nomogram demonstrated that it had satisfactory discriminative and calibration capabilities.

The OS of cancer patients is associated with their clinicopathological features. In our analysis, advanced age had worse outcomes in terms of OS mainly because elderly patients have more comorbidities that could negatively impact the survival time and influence treatment doses for low tolerability (14, 15).

Studies had different views on gender as a prognostic factor for overall survival in patients with PCL. In the previous study, female sex was an independent factor for improved survival (16). However, in our study, there was no statistically significant difference between male and female in the OS, partly because of the low incidence of PCL and the number of PCL cases we extracted in the SEER database was limited.

PCL mainly occurs in the ileocecal region with abundant lymphoid tissue, followed by the rectum. According to our study, PCL patients whose primary lymphoma site was located in the rectum had better survival than patients with disease on the right and left sides. The following possible explanations are provided. Considering that the rectum is narrower than the colon, patients are more likely to have altered bowel habits and characteristics. The tumor location is easier to identify by colonoscopy or anal digital examination, and it is easier to find and diagnose early, which may be helpful for better survival.

Our analysis detected marital status as an independent factor for improved PCL patient survival, which is the first description of the role of this factor in PCL. Marital status has been demonstrated to provide some benefits in various cancer categories (17). Previous studies showed that married patients had better overall survival than unmarried patients (including separated, divorced, widowed, and single patients) (18–20). The probable causes of differences between married and unmarried patients are as follows: 1)married patients benefit from more support, which can be beneficial to the diagnosis and treatment from their spouse, who may encourage patients to seek medical help for distressing symptoms and to accept definitive management (21) and provide tumor patients more social support when undergoing cancer treatment; 2) married patients tend to have less anxiety, stress, and depression when they are diagnosed with cancer, and several high-quality studies report that a good marriage is associated with improvements in cardiovascular, endocrine, and immune function (22, 23).

Among the clinicopathological variables, the Ann Arbor stage and histological subtypes were observed to be significantly associated with PCL patient survival. Patients with early-stage lymphoma tended to have a better prognosis, which is consistent with previous studies. Moreover, in our study, DCBCL was the most common histological type of PCL, accounting for 57.8% of the collected data, followed by MZL (20.8%), FL (10.7%), and MCL (10.6%). However, in our survival analysis, FL and MZL patients had better survival outcomes than DCBCL and MCL patients.

Colorectal lymphoma is a lymphoma that occurs in the submucosal lymphoreticular tissue of colorectal, and the tumor has heterogeneity and diversity. Colorectal lymphoma is a type of disease that easily spreads all over the body because of these widely distributed lymphoid tissues. Most scholars thought that the treatment strategies of surgery combined with radiation or chemotherapy are reasonable. Our research result showed that surgery remained the mainstay of treatment for early-stage PCL patients overtime. Radical surgery can be performed for those with localized lesions, and palliative resection can be performed for those with extensive lesions. This procedure not only removes the main tumor but also prevents complications, such as obstruction, and the subsequent need for radiotherapy and chemotherapy after surgery. Therefore, surgical resection of the lesion is an important factor that determines the survival time. In our study, compared with chemotherapy-only treatment, the combination of surgery and chemotherapy treatment prolonged the OS of PCL patients. Similarly, patients who underwent a combination of surgery and radiation had better OS than patients who received radiation alone. Moreover, the combination of surgery, radiation, and chemotherapy can significantly prolong OS. Notably, we should pay attention to the role of surgery.

Then, the predictive accuracy of the nomogram was evaluated by AUC values. In the training cohort, the 1-, 3-, 5-, and 10-year AUC values of the nomogram for OS were 0.746, 0.743, 0.757, and 0.757, respectively. In the validation cohort, the 1-, 3-, 5-, and 10-year AUCs were 0.725, 0.731, 0.754, and 0.754. This discrimination of the nomogram revealed good prognostic accuracy and clinical applicability.

The final nomogram was comprised of age, Ann Arbor stage, histology, location, marital status, and treatment, which scores the risk factors and has an effect on predicting the 1, 3, 5, and 10‐year survival probabilities of the tumor, then applies in the medical domain for clinical decision-making. The nomogram with a better ROC curve had better clinical discrimination and calibration. The nomogram can provide reliable prediction basis for PCL patients. Regarding application of our PCL nomogram, for example, consider an unmarried 24-year-old man with stage I, DCBCL histology, and right-sided disease treated with surgery only. To assess his overall survival, he scored 344 points for his age, 64 points for the location, 70 points for his marital status, 64 points for Ann Arbor stage, 64 points for tumor histology, and 57 points for treatment for a total of 663 points. The total points correspond to a 1-year survival probability of 95%, a 3-year survival probability of 92.4%, a 5-year survival probability of 89.8%, and a 10-year survival probability of 83%.

## Limitations

The limitation of our study is the incomplete information from SEER database. For example, we cannot detect specific therapeutic strategies, and it is unknown whether the different types of surgery performed or the chemotherapy regimen chosen profits to the survival improvement.

## Conclusion

We developed and validated a six-factor (age, histology, Ann Arbor stage, marital status, location, and treatment) nomogram for predicting prognosis in PCL patients. Elderly individuals and patients with advanced stage disease who were not married and had DCBCL histology exhibited poor survival. Surgery combined with other treatments had an important role in OS for PCL patients. The advantage of this study involves its exploration of related factors that affect the prognosis and survival of PCL patients and the value of treatment methods using a multidimensional nomogram to provide a theoretical basis for the treatment of colorectal lymphoma.

## Data availability statement

Publicly available datasets were analyzed in this study. This data can be found here: http://seer.cancer.gov/.

## Author contributions

QC, YF, JY, and RL contributed to conception and design. QC and YF conducted data collection and analyzed the data. QC, YF, and JY interpreted the data. QC, YF, and JY drafted the manuscript. QC, YF, JY, and RL contributed critical revision of the manuscript. All authors read and approved the final manuscript.

## Funding

This work was supported in part by the Chongqing Science and Health Joint medical scientific research project (2021MSXM283) and China Postdoctoral Science Foundation funded project (2018M633329) for RL.

## Conflict of interest

The authors declare that the research was conducted in the absence of any commercial or financial relationships that could be construed as a potential conflict of interest.

## Publisher’s note

All claims expressed in this article are solely those of the authors and do not necessarily represent those of their affiliated organizations, or those of the publisher, the editors and the reviewers. Any product that may be evaluated in this article, or claim that may be made by its manufacturer, is not guaranteed or endorsed by the publisher.
