# Supplementary figures and images for: Development and validation of a population-based prognostic nomogram for primary colorectal lymphoma patients
Source: Front Oncol. 2022 Oct 24;12:991560. doi: 10.3389/fonc.2022.991560 (PMC9638023; doi:10.3389/fonc.2022.991560)

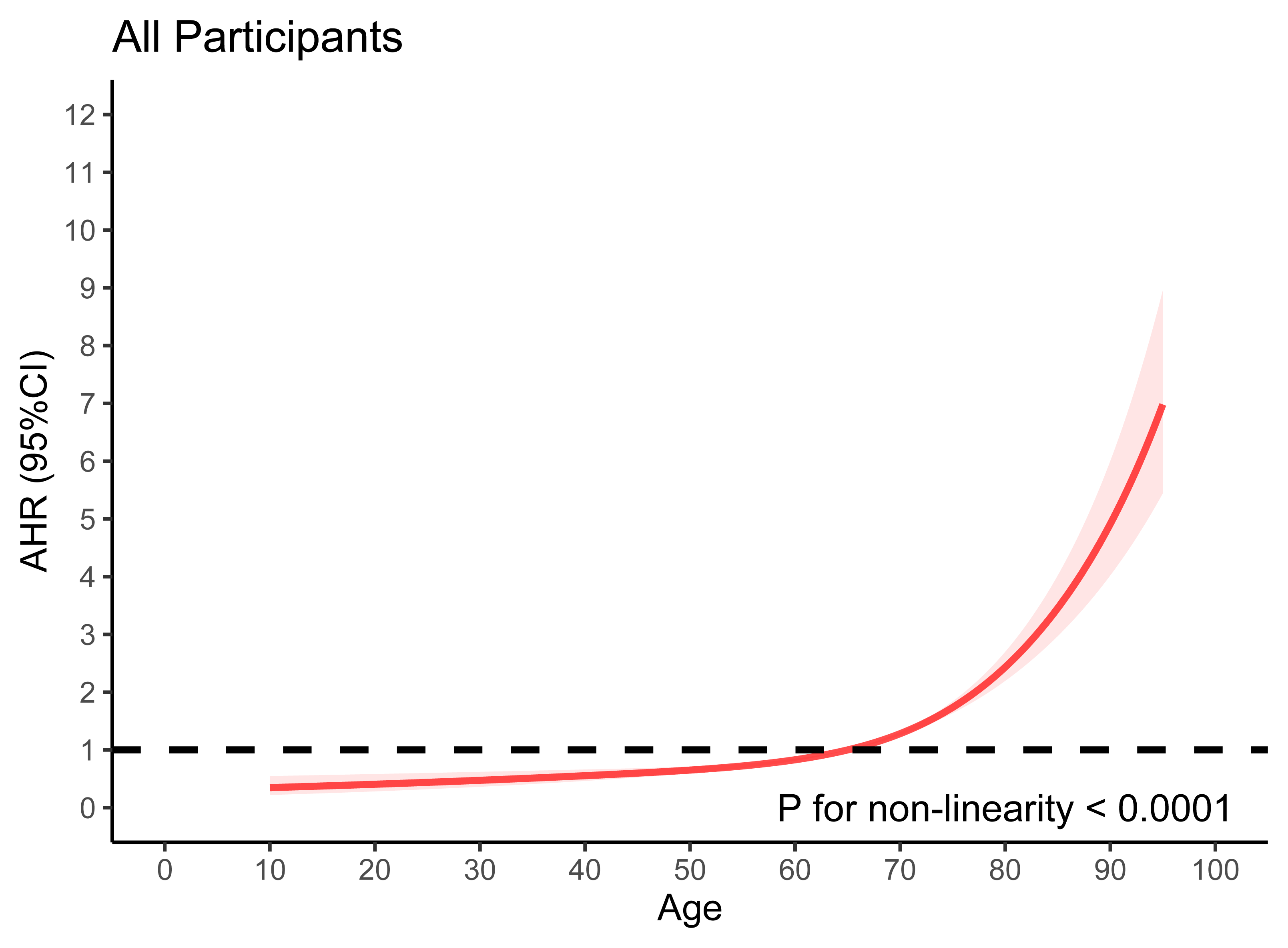

Supplement: Supplementary file 1 [file Image_1.tif]
